# Effects of a 4-Step Standard Operating Procedure for the Treatment of Fever in Patients With Acute Stroke

**DOI:** 10.3389/fneur.2021.614266

**Published:** 2021-03-04

**Authors:** Hanna Lee, Günter Hedtmann, Stefan Schwab, Rainer Kollmar

**Affiliations:** ^1^Department of Neurology, University Hospital Erlangen, Friedrich-Alexander-University Erlangen-Nürnberg (FAU), Erlangen, Germany; ^2^Department of Neurology and Neurological Intensive Care, Darmstadt Academic Teaching Hospital, Darmstadt, Germany

**Keywords:** acute stroke, standard operating procedure, fever, hyperthermia, fever treatment

## Abstract

**Background and Purpose:** Fever in the acute phase of stroke leads to an unfavorable clinical outcome and increased mortality. However, no specific form of effective fever treatment has been established, so far. We analyzed the effectiveness of our in-house standard operating procedure (SOP) of fever treatment.

**Methods:** This SOP was analyzed for a period of 33 weeks. Patients with cerebral ischemia (ischemic stroke, transient ischemic attack) or cerebral hemorrhage (intracerebral, subarachnoid) and body temperature elevation of ≥ 37.5°C within the first 6 days after admission were eligible for inclusion in the analysis. The results of SOP group, who's data have been collected prospectively were then compared with a historical control group that had been treated conventionally 1 year earlier in the same period. The data of control group have been collected in retrospect. The primary endpoint was the total duration of the fever for the first 6 days after admission to the stroke unit.

**Results:** A total of 130 patients (mean age of 78 ± 12) received 370 antipyretic interventions. Sequential application of paracetamol (*n* = 245), metamizole (*n* = 53) and calf compress (*n* = 15) led to significant reduction in body temperature. In patients who did not respond to these applications, normothermia could be achieved after infusion of the cooled saline solution. Normothermia could be achieved within 120 min in more than 90% of the cases treated by the SOP. The SOP reduced the fever duration in the 6 days significantly, from 12.2 ± 2.7 h [95% confidence interval (CI) for mean] in the control group to 3.9 ± 1.0 h (95% CI) in the SOP group (*p* < 0.001). The SOP was rated to be reasonable and effective.

**Conclusion:** Our in-house SOP is cost-efficient and effective for fever treatment in stroke patients, that can be implemented by local health care professionals.

## Introduction

Numerous clinical and experimental studies have indicated the association between elevated core body temperature and unfavorable clinical outcome in patients with acute stroke (1–4). In experimental stroke, a temperature decrease of 1–2°C in the intraischemic or postischemic brain has been found to be neuroprotective, while a slight temperature increase significantly worsens the outcome (5). A clinical study reported the relative risk of increasement of poor outcome by a factor of 2.2 for 1°C increase in body temperature (6). A negative effect of hyperthermia on functional outcome was demonstrated even in patients treated with intravenous recombinant tissue plasminogen activator (rt-PA). Hyperthermia within 3 days after rt-PA is associated with poor functional prognosis and survival outcome in patients with acute cerebral infarction (7). Similar effects of body temperature have been in patients with intracerebral hemorrhage (8).

Although paracetamol is used frequently in pyretic stroke patients, it showed limited effect on body temperature and no effect on the functional outcome compared with placebo (9). Thus, the current recommendations for fever treatment in stroke are rather unspecific and have a questionable impact on fever burden and outcome (10).

Some non-pharmacological techniques to reduce fever include physical methods such as high flow transnasal air or external cooling devices (11). They are effective, but highly expensive and require sometimes invasive procedures. Most importantly, they are associated with side effects such as shivering, which warrant more aggressive pharmacological management (12).

An in-house standard operating procedure (SOP) was previously presented in a pilot study, which was applied over a relatively short period of time and demonstrated its satisfactory antipyretic effect in stroke patients (13). This SOP included a sequence of 4 pharmacological and physical interventions, instead of a single-drug approach. Based on the former results, we have expanded our clinical investigation with an increased number of patients over a longer period of time.

We report the effects of our SOP regarding frequency of fever, fever duration and neurological outcome. These results were compared to a historical control group which has been treated without adherence to a standardized protocol.

## Methods

### Standard Operating Procedure

**Figure 1:**
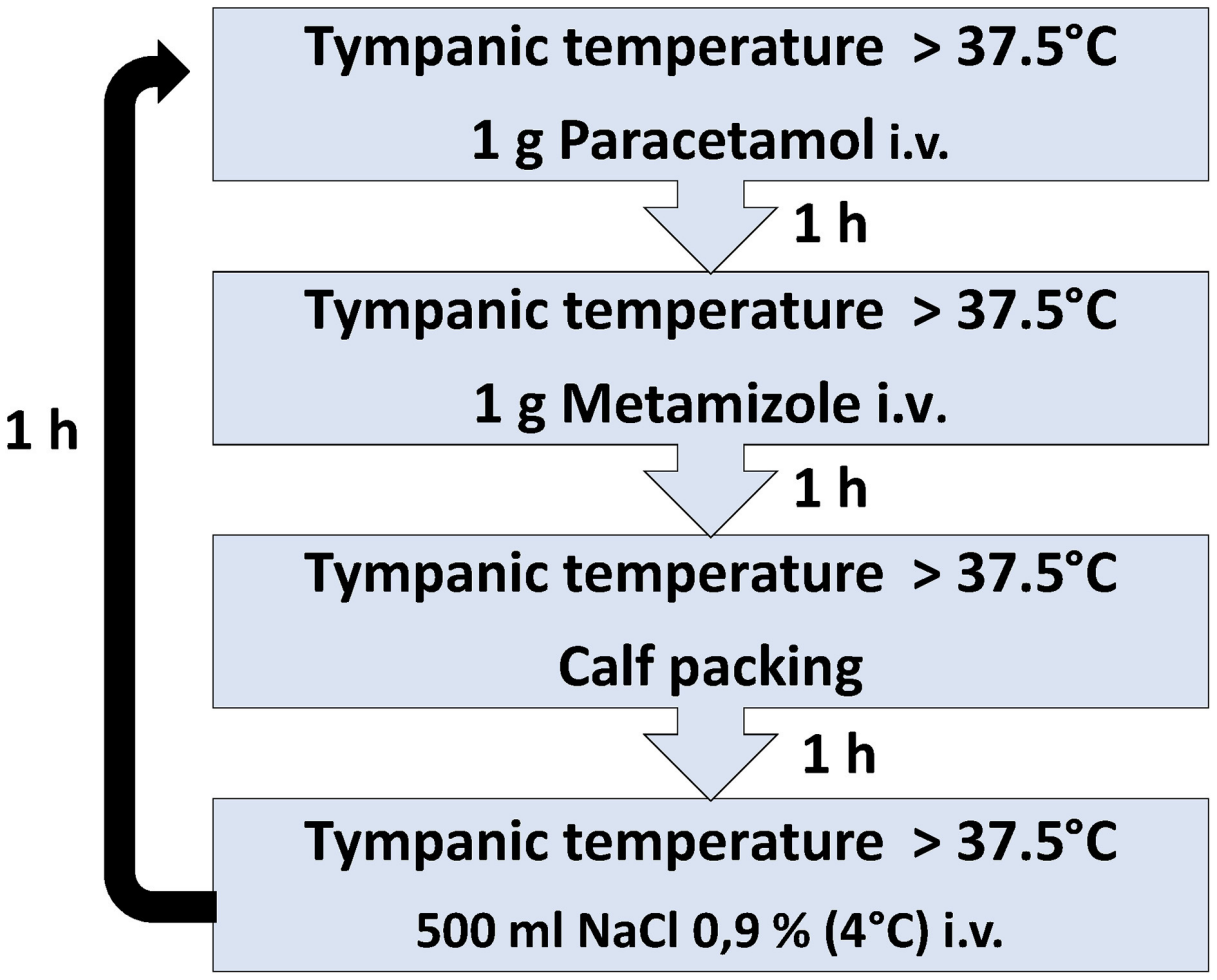
The SOP includes a sequence of four antipyretic interventions with close measurement of body temperature.

This 4-stage SOP contains a sequence of 4 pharmacological and physical interventions instead of a monotherapy (Figure 1). The SOP was implemented in April 2017 and represents the local standard of care since this timepoint. The data included for this investigation of the SOP were analyzed for a period of 33 weeks, from May to December 2017. This SOP was eligible for the patients with brain ischemia (ischemic stroke, transient ischemic attack) or cerebral hemorrhage (intracerebral, subarachnoid) and body temperature elevation of ≥37.5°C in the first 6 days after admission. They were diagnosed by brain imaging and clinical examination. The primary endpoint was defined as the total duration of fever during the first 6 days after admission to the stroke unit.

In stage 1, patients are treated by a single infusion of 1.000 mg paracetamol and, in stage 2, by a single infusion of 1.000 mg metamizole, through a peripheral or central intravenous catheter. In cases of limited effects of these antipyretics, external cooling by calf packing is applied for 60 min at stage 3 and finally an intravenous application of 500 ml of cooled (4°C) saline solution (0.9% NaCl) is administered via 30 min at stage 4. The last stage is suitable for patients without clinical signs of congestive heart failure or dyspnea (NYHA III or IV). Body temperature measurement is performed 60 min after each stage. Body temperature >37.5°C after completion of the final step results in return to stage 1. The treatment is limited to a maximum of 4 complete cycles within 24 h. All patients receive continuous non-invasive cardiac monitoring, including ECG, oxygen saturation, respiratory rate, heart rate and arterial blood pressure. Routine laboratory tests are performed on admission (gamma -glutamyl transpeptidase, glutamic oxaloacetic transaminase, glutamic acid pyruvate transaminase, C-reactive protein (CRP), blood count and electrolytes) at least once during the following 6 days in the Stroke unit. In both the SOP and control group, the screening for body temperature was performed with an electronic tympanic thermometer (Genius 2 Tympanic Thermometer; Tyco Healthcare Group, Mansfield, Massachusetts, USA) at 4-h intervals. In case of fever the temperature was measured every hour in both groups.

The body temperature before and 60 min after each stage of antipyretic intervention as well as the duration of the body temperature of ≥ 37.5° C per day were measured. We recorded the time from the initiation of the antipyretic treatment to the attainment of normothermia or early relapse of fever as well. Here, “Early relapse” is defined as temperature increase within 24 h after achieving normothermia. Finally, the length of stay on the stroke unit and the NIHSS score points (National Institutes of Health Stroke Scale) on discharge were documented.

### Control Group

The historical control group consisted of 74 patients who had been treated at the Stroke Unit of Darmstadt Academic Hospital for 16 weeks, from May to September 2016, 1 year earlier in the same period, which is exactly half of the time period of the study group. The patients were diagnosed with brain ischemia (ischemic stroke, transient ischemic attack) or cerebral hemorrhage (intracerebral, subarachnoid) and with a body temperature of ≥ 37.5°C within the first 6 days after admission. Fever in the control group was treated according to the recommendations of the European Stroke Organization guidelines without application of the formerly described SOP (14, 15).

### Data Management and Evaluation

Every single implementation of the SOP in patients with body temperature of ≥ 37.5°C was recorded daily by the nursing staff and by physicians of the Stroke Unit. The data of SOP group were collected prospectively, while the data of control group were collected retrospectively. Data was investigated after pseudonymization of the patient chart.

Statistical evaluations were created with R Version 3.5.2 (16). For normally distributed data, mean values and standard deviations were calculated. We assumed that populations had different variances, and used one-tailed Welch's *t*-test (17). For count data, absolute and relative frequencies are given.

## Results

### Patient Characteristics

*N* = 1,034 patients in total were treated at the Stroke Unit of the Darmstadt Academic Hospital during the 33-week study. 767 patients of them were diagnosed with “acute ischemic stroke,” “intracerebral hemorrhage,” “transient ischemic attack” or “subarachnoid hemorrhage.” 130 (16.9%) of 767 patients had a body temperature of ≥ 37.5°C within the first 6 days after admission and were included in the study. A total of *n* = 370 antipyretic interventions were applied. All patients were Caucasians; the mean age was 78 ± 12 years, 55% were female. Mean NIHSS score on first day was 9.25 ± 7.11. “Acute ischemic stroke” was diagnosed in 107 patients (82%), “transient ischemic attack” in 10 patients (8%), “intracerebral hemorrhage” in 10 patients (8%), “subarachnoid bleeding” in 2 patients (2%) and “venous sinus thrombosis” in 0 patients (0%).

Table 1 shows the patient characteristics of the SOP group and the control group including age, sex and NIHSS score on admission and discharge.

**Table 1:** Baseline characteristics of patients.

	**SOP group *n* = 130**	**Control group *n* = 74**	*P*-value^*^
**Clinical features**			
Age, years (±SD)	78 ± 12	76 ± 15	0.426
Females, *n* (%)	71 (55)	41 (55)	1
Median NIHSS day 1 (±SD)	9.25 ± 7.11	6.78 ± 6.34	0.016
Median NIHSS on day of discharge (±SD)	6.34 ± 6.62	5.91 ± 7.50	0.68
White blood cell count at baseline >10.000/μL, *n* (%)	43(33)	16 (22)	0.115
CRP at baseline > 5 mg/L, *n* (%)	62 (48)	37 (50)	0.864
GOT at baseline, U/L (±SD)	36.7 ± 42.3	27.3 ± 14.3	0.023
GPT at baseline, U/L (±SD)	24.1 ± 18.2	21.6 ± 18.0	0.346
GGT at baseline, U/L (±SD)	55.2 ± 73.4	60.4 ± 106.1	0.707
**Cardiovascular risk factor profile**			
Atrial fibrillation, *n* (%)	55 (42)	22 (30)	0.103
Arterial hypertension, *n* (%)	109 (84)	68 (92)	0.157
Hyperlipidemia, *n* (%)	82 (63)	48 (65)	0.917
Active smoking, *n* (%)	7 (5)	4 (5)	1
Diabetes Mellitus, *n* (%)	50 (38)	25 (34)	0.606
Persistent foramen ovale, *n* (%)	7 (5)	4 (5)	1
History of stroke, *n* (%)	31 (24)	21 (28)	0.584
Coronary artery disease, *n* (%)	21 (16)	12 (16)	1
History of myocardial infarction, *n* (%)	9 (7)	4 (5)	0.773
Peripheral artery disease, *n* (%)	4 (3)	4 (5)	0.465
**Type of cerebrovascular disease**			
Transient ischemic attack, *n* (%)	10 (8)	8 (11)	0.618
Ischemic stroke, *n* (%)	107 (82)	56 (76)	0.340
Intracerebral hemorrhage, *n* (%)	10 (8)	6 (8)	1
Subarachnoid hemorrhage, *n* (%)	2 (2)	4 (5)	0.193
**Detected infections**			
Pneumonia, *n* (%)	27 (21)	9 (12)	0.174
Urinary tract infection, *n* (%)	18 (14)	11 (15)	1
Other infection, *n* (%)	15 (12)	15 (20)	0.194

*NIHSS, National Institutes of Health Stroke Scale; CRP, c-reactive protein; GOT, glutamic oxaloacetic transaminase; GPT, glutamic pyruvate transaminase; GGT, gamma-glutamyl transpeptidase. SD, standard deviation*.

* *p-values from t-test for continuous variables, Pearson's Chi-squared test with Yates' continuity correction for categorical data and Fisher's Exact Test for Count Data in case of counts lower than five*.

### Prevalence of Fever and Infections

There was no significant difference in prevalence of fever between the SOP and control groups (Table 2A). The number of days on which fever was overall detected showed no significant difference between SOP and control group. In most cases fever started on day 1 and 2 after admission. No patient developed the first fever period on day 5 and 6.

**Table 2A:** Prevalence of fever with number of patients in SOP and control groups.

	**Total Duration of first fever (days)**				
	1 day	2 days	3 days	4 days	5 days
**SOP group (*****n*****, %)**	64 (50%)	36 (28%)	21 (16%)	5 (4%)	4 (3%)
**Control group (*****n*****, %)**	34 (46%)	22 (30%)	8 (11%)	8 (11%)	2 (3%)

Table 2B shows the contribution of the first fever onset. In the control group, there were slightly more patients developing fever on day 1 than on day 2. In the SOP group, fever occurred on day 2 in most patients.

**Table 2B:** Days of fever onset.

**Onset of fever**	**SOP group (*n*, %)**	**Control group (*n*, %)**
Day 1	46 (35.4%)	30 (40.5%)
Day 2	59 (45.4%)	28 (37.8%)
Day 3	23 (17.7%)	13 (17.6%)
Day 4	2 (1.5%)	3 (4.1%)

An increase in body temperature to ≥ 37.5°C with the indication for antipyretic treatment was detected for 370 times in SOP group (Table 3). On admission, 48% of patients (*n* = 62) had CRP values >5 mg/dl. Thirty three percent of patients (*n* = 43) had leukocyte counts >10.000/μl. During treatment, an infectious focus was identified in 46% of patients (*n* = 60) (Table 1).

**Table 3:** Effects of sequential antipyretic interventions (*n* = 370) in 130 febrile patients.

**Antipyretic intervention**	**Numbers of intervention, n**	**Median temperature before intervention, ^**°**^C (±SD)**	**Median temperature after 60 min ^**°**^C (±SD)**	**Normothermia achieved, *n* (%)**	**Early relapse (<24 h), *n* (%)**	**Median time to relapse, h (±SD)**	**Antibiotic treatment, *n* (%)**
P1	225	38.0 ± 0.3	37.2 ± 2.1	168 (75)	46 (27)	2.1 ± 4.8	38 (17)
M1	49	38.2 ± 0.5	37.6 ± 0.5	27 (55)	12 (44)	2.4 ± 4.8	6 (12)
CP1	15	38.4 ± 0.6	38.2 ± 0.6	2 (13)	1 (50)	0.9 ± 3.2	3 (20)
CS1	55	38.1 ± 0.4	37.5± 0.7	34 (62)	12 (35)	2.2 ± 4.5	8 (15)
P2	20	38.0 ± 0.4	37.5 ± 0.4	13 (65)	7 (54)	2.8 ± 4.2	2 (10)
M2	4	38.0 ± 0.3	37.5 ± 0.4	3 (75)	1 (33)	3.0 ± 6.0	0 (0)
CS2	2	37.8 ± 0.1	37.4 ± 0.1	2 (100)	1 (50)	6.0 ± 8.5	1 (50)
CP2	0	0	0	0	0	0	0

*P1, Paracetamol first bolus; M1, Metamizole first bolus; CP1, first application of calf packing; CS1, first infusion of cooled saline; P2, Paracetamol second bolus; M2, Metamizole second bolus; CP2, second application od calf packing; CS2, second infusion of cooled saline. Data are means ± standard deviation and absolute number N and relative frequency in %, respectively*.

The percentage of patients in the control group with elevated CRP levels on admission was 50% (*n*=37), leukocytosis was 22% (*n* = 16). A focus of infection could be identified in 47% of patients (*n* = 35) (Table 1).

### Antipyretic Effects of the SOP

Paracetamol was given the most (*n* = 225), followed by metamizole (*n* = 49), calf packing (*n* = 15) and infusion of cooled saline (*n* = 55). A significant reduction of the mean body temperature measured 60 min after each intervention could be observed (Table 3). A tendency toward a decrease body temperature was observed after infusion of cooled saline (*n*=55). Normothermia was achieved in 62% of cases after infusion of cooled saline, which represents the last step of the SOP.

Normothermia could be achieved by application of the second bolus of paracetamol in 13 of the remaining 20 patients who still had a body temperature of >37.5°C after completion of first round of the SOP.

After the patients reached normothermia, early relapse occurred in 54% of cases within 24 h. The shortest median time to early relapse was 0.9 ± 3.2 after first application of calf packing, while the longest median time to early relapse was 6.0 ± 8.5 h after second infusion of cooled saline. The mean NIHSS score of the SOP group on discharge was 6.34 ± 6.62 (the mean NIHSS score on admission was 9.25 ± 7.11), while the mean NIHSS score of the control group was 5.91 ± 7.50 (the mean NIHSS score on admission was 6.78 ± 6.34).

**Figure 2:**
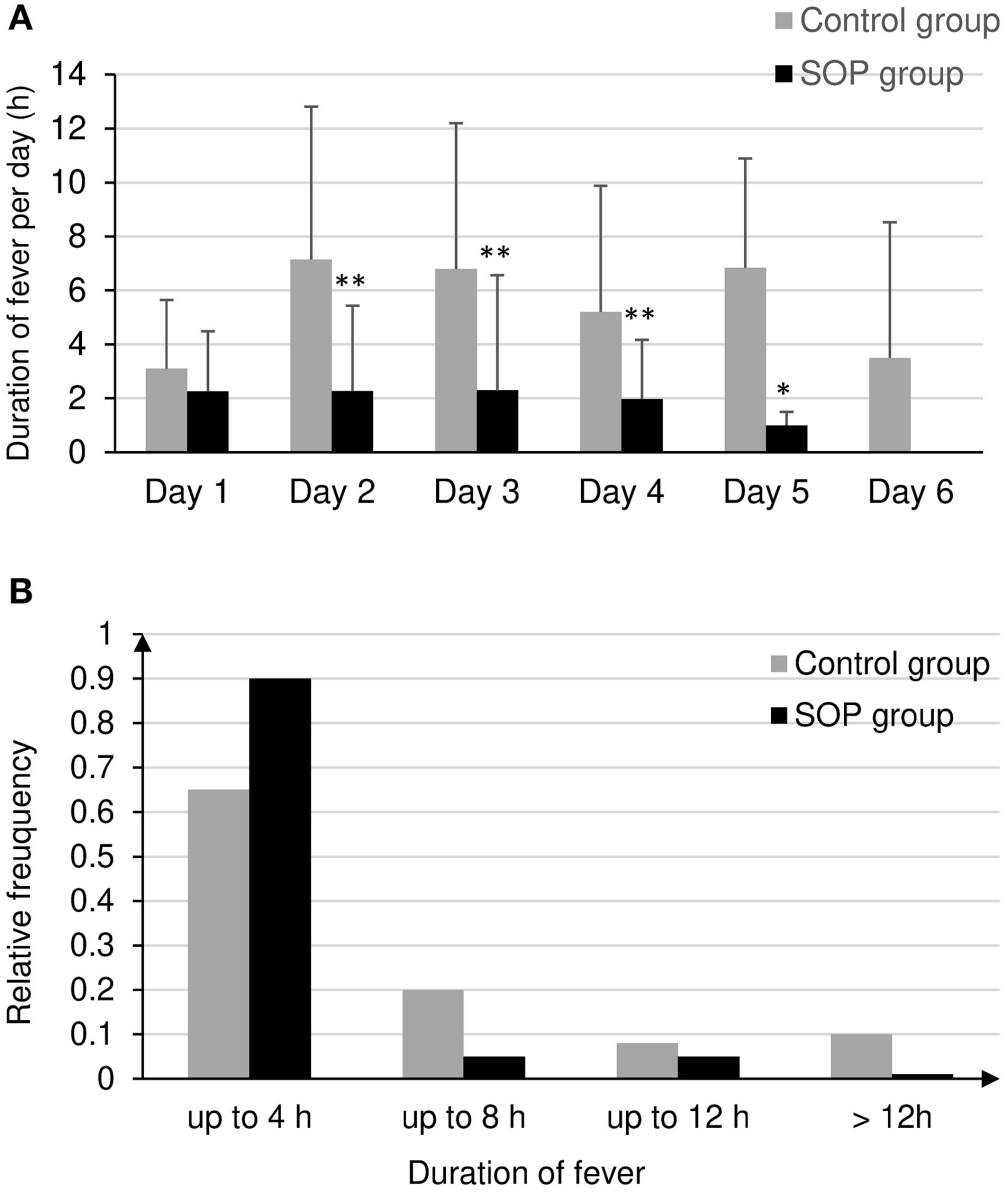
**(A)** Mean duration of body temperature >37.5°C in hours per day after admission to the stroke unit in the SOP group and in the control group, *p < 0.01, **p < 0.001 by *t* test. **(B)** Distribution of duration of first fever in the SOP and control group.

Most importantly, the SOP had a significant effect on primary endpoint. The primary endpoint was defined as the total duration of fever during 6 days after admission. The fever duration has been reduced significantly, from 12.2 ± 2.7 h (95% confidence interval for mean) in the control group to 3.9 ± 1.0 h (95% CI) in the SOP group (*p* < 0.001). We could observe a mean daily duration of fever of 2 ± 0.6 h in the SOP group, while it was 5.4 ± 1.8 h in the control group (Figure 2A). The mean duration of body temperature >37.5°C in the SOP group was considerably shorter in all days compared to the control group. No patients in the SOP group had a body temperature >37.5°C on day 6.

Figure 2B shows the difference in the duration of the first fever between study and control groups in a 4-h interval. Fever lasted significantly longer in the control group than in the SOP group. In 90% of the patients in the control group, fever lasted longer than 12 h. In 90% of the patients in the SOP group the fever lasted up to 4 h (Figure 2B).

### Drug Administration

The maximum daily dose of 4 g/day was never exceeded during paracetamol administration in both OP and control group. The mean daily doses were similar in the control group with 1.03 ± 0.49 g/day and SOP group with 1.06 ± 0.52 g/day. Similar results were observed for metamizole administration. The mean daily dose of metamizole in the control group was 0.83 ± 0.41 g/day and 1.53 ± 1.10 g/day in SOP group.

A total of 60 patients (46%) in the SOP group underwent antibiotic treatment during the treatment on the stroke unit. A total of 35 patients (47%) in the control group underwent antibiotic treatment during the treatment on the stroke unit.

### Safety Aspects

Eight patients in the SOP group (6.1%) and five patients in the control group (6.7%) died during their stay in hospital. The admission diagnosis in nine patients was ischemic stroke, in four patients intracerebral bleeding. Eight patients died of multimorbidity and palliative therapy, one patient of sepsis caused by endocarditis, two patients of malignant infarction in palliative approach and two other patients of pulmonary embolism and pneumonia.

## Discussion

In this study we investigated the effects of an in-house SOP for temperature treatment, which is implemented in an acute phase in patients with acute ischemic stroke or cerebral hemorrhage. This in-house SOP includes a sequence of pharmacological and physical interventions, since we considered an escalation therapy to be more effective for fever reduction than monotherapy. A few years ago, a pilot study on this SOP indicated significant effects on fever after acute stroke (13). In more than 90% of cases treated per SOP, normothermia was achieved within 120 min. Compared to conventional treatment, fever burden was significantly lower within the first 4 days after admission (*p* < 0.001). However, the number of patients was rather small and there has been no follow-up investigation on this SOP so far. Therefore, we analyzed the effects of a newly introduced SOP in our department with an increased number of patients and an extended period of time compared to the former investigation. A total of 130 patients treated by the SOP over a total period of 33 weeks were included in the analysis. These patients had developed a body temperature of ≥ 37.5°C in the first 6 days after admission to the stroke unit. A historical control group consisted of 74 patients who had been treated 1 year earlier according to the recommendations of the European Stroke Organization guidelines (14). The incidence of fever was similar between SOP and control group. However, there was a significant difference in the duration of fever periods in hours between both groups. The sequential application of paracetamol, metamizole, calf packing and infusion of cooled saline in the SOP group resulted in a significant reduction in body temperature 1 h after each implementation. This showed a remarkable difference in the fever burden compared to the historical control group treated with a monotherapy of paracetamol. The period of fever in the SOP group was considerably shorter at each day compared to the control group. Paracetamol and metamizole never exceeded the allowed maximum daily dose in both SOP and control group.

Previous studies investigated the effects of paracetamol and other medications as a monotherapy on neurological outcome and fever after acute stroke (9, 10, 18–21). None of them showed improved neurological outcome followed by antipyretic medication. This is also the case in our investigation. However, we have been able to show that fever as the major target parameter could be significantly lowered in a meaningful time period. The PAIS study showed that treatment with a daily dose of 6,000 mg of paracetamol lowered body temperature by 0.3°C (95% CI: 0.1–0.5) (19). This minimal impact on fever could easily explain why the neurological outcome could not be improved by the named approach. In contrast, our SOP could represent a powerful strategy for a large randomized trial to investigate effect on effective fever treatment on neurological outcome, since our SOP influences body temperature effectively.

Non-pharmacologic techniques often show reduction of fever. For example, transnasal cooling has been demonstrated its effectivity with temperature decrease by an average of 1.21°C (SD 0.46) within 1 h (11). However the side effects such as shivering, discomfort and steep increases in blood pressure raise serious concerns regarding the safety of its use in stroke patients (11, 12). In our study, we report efficacy of sequential use of two pharmacological and two physical treatments without any vital side effect, such as arterial hypertension or shivering. In addition, the use of SOP does not require invasive monitoring or procedures, which can be therefore considered to be more feasible and cost-efficient.

One limitation of our study is the lack of permanent temperature monitoring. Each temperature measurement in the patients was carried out manually using a tympanic thermometer. This could delay the detection of the fever manifestation, although the temperature was measured by the nursing staff every 4 h. For more accurate temperature measurement, permanent temperature monitoring should be used (22). The lack of precision of measurement with a tympanic thermometer was also cited in a previous study (13). The rate of calf packing was less compared to cold infusions indicating that nursing stuff favors short application modes such as infusions. Therefore, further studies should focus on sequences of medication and iv cooling and potentially not include time consuming calf packing.

Moreover, this observation is not a randomized controlled study and therefore data has to be interpreted with caution. Temperature-lowering effect of the antibiotic treatment was already described in an earlier study (23). Prophylactic or early calculated antibiotic treatment must also be discussed further.

## Conclusion

The presented 4-stage SOP is a cost efficient and effective method for fever treatment in stroke patients, that can be easily implemented in everyday clinical practice. Further studies should investigate this approach in a randomized fashion.

## Data Availability Statement

The raw data supporting the conclusions of this article will be made available by the authors, without undue reservation.

## Ethics Statement

The studies involving human participants were reviewed and approved by Ethics Committee of the State Chamber of Physicians of Hesse in Germany. Written informed consent for participation was not required for this study in accordance with the national legislation and the institutional requirements.

## Author Contributions

RK contributed to the study design. HL performed data acquisition and analysis. RK, GH, and SS supervised the research. HL and RK wrote the article. SS and GH approved the final manuscript. All authors contributed to the article and approved the submitted version.

## Conflict of Interest

The authors declare that the research was conducted in the absence of any commercial or financial relationships that could be construed as a potential conflict of interest.
